# Supplementary material for: Chromothripsis during telomere crisis is independent of NHEJ, and consistent with a replicative origin
Source: Genome Res. 2019 May;29(5):737–49. doi: 10.1101/gr.240705.118 (PMC6499312; doi:10.1101/gr.240705.118)
Supplement: Supplemental Material [file supp_gr.240705.118_Supplemental_file_1.zip › contigs/annotated_contigs/DB45/contig.2.DB45_length_576_mean_cov_5.72916666667.docx]

**DB45_length_576_mean_cov_5.72916666667**

```
AAATAAATGTATAGCTATACAGGAGGAAGAATGTGGTAGCAGGAGAGTACAGTAGTTTCTCATTATTTGTGGTTTCACTTTTTGAGGTT
 >chr20:58448441-58448723 - E=5e-158
TCAGTTACCTACAGTCAACTGTGGTCTGATAATATTAAATAAGAAATTGCAGAAATAAACAATTCATATGTTTTACATTGTGTGTAATT

CTGAATAGTGTGATGAAATATTGAGCCATCCACTCTGTCCCTCCTGGGACGTGAATCAACCCTTTGTCCAGAGTATCCATGCTGCTGTA

TACCCTACCTGC|CTG|GAGCATAGAAACTGTACTAGCAGGGATCCTTATCTACAGGTTTTGGTCATCAAAGTAATAATCCACTTGCCT
             >chr20:58444980-58445277 - E=4e-167
AAGGATTTTTTATTTTTAGTTATGAATTGGAATTGAATTTTTGCAGTGAATTAGTAGTTTTGCAGTTTTGCTTTAATGTTTCTTATGTT

CAACTCTTTTTGAAGTGGGGGGCTTTGAACAATTTTGTAACTTCTTATTGGGTTTTTGTATGTCTTCATTCATAGCCCAGACATACTCC

AACTAAGAGTAATACTATTGTAAATAGAAAAAAAATAAGTTCTC
```
